# A systematic review and meta-analysis assessing the use of tranexamic acid (TXA) in acute gastrointestinal bleeding

**DOI:** 10.1007/s11845-023-03517-0

**Published:** 2023-10-04

**Authors:** Oisín O’Donnell, Clodagh Gallagher, Matthew G. Davey, Jonathan Coulter, Mark Regan

**Affiliations:** 1 Department of General and Colorectal Surgery, Galway University Hospitals, Saolta University Health Care Group, Newcastle Road, Galway, H91YR71 Ireland; 2 University of Limerick, Sreelane, Castletroy, Co., Limerick, V94 T9PX Ireland; 3 The National University of Ireland Galway, University Rd, Galway, H91TK33 Ireland; 4 School of Postgraduate Studies, Royal College of Surgeon in Ireland, Dublin, D02YN77 Ireland

**Keywords:** Gastrointestinal bleeding, LGIB, Systematic review and meta-analysis, Tranexamic acid, TXA, UGIB

## Abstract

**Introduction:**

Gastrointestinal bleeding results in significant morbidity, cost and mortality. TXA, an antifibrinolytic agent, has been proposed to reduce mortality; however, many studies report conflicting results.

**Methods:**

The aim of the study was to perform the first systematic review and meta-analysis of RCTs to evaluate the efficacy TXA for both upper and lower gastrointestinal bleeding. This was performed per PRISMA guidelines. PubMed, EMBASE, Cochrane and Scopus databases were searched for RCTs. Dichotomous variables were pooled as risk ratios (RR) with 95% confidence intervals (CI) using the MH method with random effects modelling.

**Results:**

Fourteen RCTs were identified with 14,338 patients and mean age of 58.4 years. 34.9% (*n* = 5008) were female and 65.1% (*n* = 9330) male. There was no significant difference in mortality between TXA and placebo (RR 0.86 95% CI (0.74 to 1.00), *P*: 0.05). The secondary outcomes, similarly, did not yield significant results. These included rebleeding, need for surgical intervention (RR: 0.75 95% CI (0.53, 1.07)), endoscopic intervention (RR: 0.92 95% CI (0.70, 1.22)), transfusion requirement (RR: 1.01 95% CI (0.94, 10.7)) and length of stay (RR: 0.03 95% CI (− 0.03, 0.08)). There was no increased risk of VTE, RR: 1.29 95% CI (0.53, 3.16). One trial (*n* = 12,009) reported an increased risk of seizure in the TXA group, RR: 1.73 95% CI (1.03–2.93).

**Conclusion:**

TXA does not reduce mortality in patients with acute upper or lower gastrointestinal bleeding and may confer an increased risk of seizures. The authors do not recommend the use of TXA in acute gastrointestinal bleeding.

## Introduction

Gastrointestinal (GI) bleeding is a common presenting complaint of patients arriving to the Emergency Department (ED). Upper gastrointestinal bleeding (UGIB) incidence estimates range between 50 and 100 persons per 100,000 per year. Lower GI bleeding (LGIB) presents less frequently, with approximately 20 per 100,000 per year requiring admission in the USA [1].

UGIB has a mortality rate of between 2 and 14% [2]. Although UGIB presents 2–5 times more frequently, LGIB has a higher mortality rate. One US study showed a mortality rate of 3.12% in LGIB compared to 2.33% in UGIB [3]. Overall, the mortality rate in gastrointestinal bleeding has remained static in the last 50 years, as per NICE guidelines report in 2014, despite medical and surgical advances [4]. This is a concern.

Tranexamic acid (TXA) was developed by Shosuke and Utako Okamoto in Japan in 1965 [5]. TXA is a well-defined synthetic analogue of the amino acid lysine. It reversibly binds lysine receptor sites on plasminogen, the precursor to plasmin formation, an enzyme responsible for clot breakdown. The binding of TXA to plasminogen prevents its conversion to plasmin and therefore reduces the fibrinolytic effects of plasmin.

Both trauma and surgery trigger similar haemostatic responses including fibrinolysis (clot degradation), and in extreme cases, hyperfibrinolysis. Antifibrinolytic agents counteract this by inhibiting clot breakdown by targeting plasmin [6]. Additionally TXA is hypothesised to have an anti-inflammatory effect to counteract the pro-inflammatory properties of plasmin [6].

In 1967, Cox et al. hypothesised that gastric hyperfibrinolysis may be a key factor in inducing rebleeding in gastric and duodenal ulcers. They suggested that antifibrinolytic agents (epsilon-aminocaproic acid (EACA) and TXA) may be theoretically useful in preventing rebleeding and therefore mortality [7]. Nilsson et al. further supported this with a study on the gastric secretions of 39 patients in 1975. It showed that patients with peptic ulcer disease and haemorrhagic gastro-duodenitis had higher levels of fibrinolytic activity [8].

A number of large randomised control trials (RCTs), including CRASH-2 [6], WOMAN [9] and CRASH-3 [10], assessing the use of TXA have emerged as safe and beneficial in reducing mortality in bleeding trauma, obstetric and head injury patients, respectively. TXA is also commonly used in surgery, particularly orthopaedic surgery to reduce operative blood loss [11].

However, the literature in relation to the use of TXA in gastrointestinal bleeding has not conclusively shown any benefit. A 2014 Cochrane review by Bennett et al. found that TXA appeared to have a beneficial effect on mortality in patients with UGIB. However, in 2021, a large (*n* = 12,009) multicentre international double-blinded randomised control trial (HALT-IT) found that TXA did not reduce mortality and conferred an increased risk of VTE events. The recommendation from the HALT-IT trial was that TXA should not be used in management of GI bleeding [12].

Since the publication of HALT-IT, two notable systematic reviews and meta-analyses looking into TXA in UGIB were performed by Burke et al. and Kamal et al. They included data from HALT-IT [13, 14]. Burke et al. included 8 RCTs and concluded that while TXA did not have a beneficial effect on mortality, it reduces rebleeding and need for surgery [13]. Kamal et al. included 12 RCTs and concluded that TXA did not improve mortality or other secondary outcomes and conferred an increased risk of venous thromboembolism (VTE). However, this increased risk of VTE only became statistically significant when assessing the subgroup of high-dose TXA vs placebo and the association between TXA at any dose and VTE was not statistically significant [14].

When it comes to complications associated with TXA, specifically increased risk of VTE, there is contradicting information in the literature. From a theoretical perspective, studies in animal models have shown increased thrombus formation in a dose-dependent manner with administration of TXA [15]. However, large RCTs have shown that TXA does not increase the risk of VTE [6], while others show new evidence to dispute this [12].

The aim of this research was to systematically compile and evaluate all of the available RCT data on TXA in GI bleeding and ascertain whether it is safe and/or beneficial in reducing mortality and to review other outcomes. Specifically the aim of this review was to build on previous systematic reviews and meta-analyses by Burke et al. and Kamal et al. [13, 14] by including trials assessing TXA in lower gastrointestinal bleeding along with UGIB, similar to the approach taken in the HALT-IT trial. This review is the first to incorporate both lower and upper gastrointestinal bleeding into the same systematic review and meta-analysis. We also wanted to evaluate whether the increased risk of VTE seen in the HALT-IT trial would be confirmed by meta-analysis including other trials assessing TXA in GI bleeding [12].

## Methods

The systematic review and meta-analysis of published RCTs was conducted in accordance with the PRISMA (Preferred Reporting Items for Systematic Reviews and Meta-Analyses) guidelines [16]. As the research review pertains to previously published clinical trials, it does not require further consent of patients involved, and, in-keeping with local institution review board, does not require ethical approval. All of the information in the trials was anonymised and, as such, there will be no sensitive patient information requiring storage and is therefore in compliance with General Data Protection Regulation (GDPR) guidelines. All of the aforementioned study authors have contributed to the formulation of the study protocol which was subsequently registered on PROSPERO on 8th March 2022, Reference No. CRD42022308878 [17].

### PICO (Population, Intervention, Comparison and Outcome) tool

The PICO tool [18] was utilised to formulate the clinical research question, see Table 1.

**Table 1:** Population, Intervention, Control and Outcome (PICO) table

Population/patients with condition	Adult patients with gastrointestinal haemorrhage
Intervention	Tranexamic acid by any route
Control	Placebo
Outcome	Mortality

### Aim and outcomes

The aim of this review was to assess the efficacy and safety of the use of TXA in acute gastrointestinal bleeding. The primary outcome was the effect of TXA on mortality in patients with gastrointestinal haemorrhage when compared with placebo. The secondary outcomes are detailed in Table 2.

**Table 2:** Table of secondary outcomes

**Secondary outcomes**
Need for intervention (endoscopic, surgical and radiological)
Need for therapeutic endoscopic intervention
Need for surgical intervention
Rebleeding rate
Transfusion requirement
Volume of transfusion
Overall length of stay (LOS)
ICU length of stay
Venous thromboembolic events (DVT, PE)
Arterial thromboembolic events (e.g. MI, stroke)
Other adverse effects of tranexamic acid

### Search strategy

The detailed search criteria for the review was composed by converting key words into Medical Subject Headings (MeSH) terms. The search terms “Gastrointestinal Hemorrhage” [Mesh] AND “Tranexamic Acid” [Mesh] were then used to perform an electronic search of MEDLINE (PubMed), EMBASE, Cochrane (CENTRAL) and Scopus databases on 31st March 2022. Searches of clinical trial registries, EU Clinical Trials Register and ClinicalTrials.gov, were also performed. All published studies regardless of publishing language were included, provided an English translation was available. There were no restrictions based on the year of publication. The retrieved studies had duplicates removed and then the titles, abstracts and full texts were screened to identify the studies for review.

### Inclusion and exclusion criteria

The identified studies were considered if the inclusion criteria, see Table 3, was met. Studies were omitted based on the exclusion criteria, see Table 3.

**Table 3:** Inclusion and exclusion criteria table

**Inclusion criteria**	**Exclusion criteria**
Randomised controlled clinical trials	Paediatric patients
Adult patients aged 18 years or older	Non-anonymised patient information
Suspected, endoscopically, surgically or radiologically verified gastrointestinal bleeding	Non-randomised or crossover trials

### Quality assurance and quality control

The literature search was independently conducted by two reviewers using the predetermined search strategy to identify the list of trials. Duplicates were excluded, then screening of titles, abstracts and full texts was performed and application of the inclusion and exclusion criteria was assessed independently. This helped to ensure compliance with systematic review process and independent concordance of the relevant studies prior to data extraction.

### Data extraction

After independent identification of studies to be included, 14 suitable randomised control trials were identified. The following data points were extracted, where available, from the individual studies: 1. Manuscript title, 2. Lead author, 3. Year of publication, 4. Country of research facility, 5. Single/multicentre study, 6. Blinding implemented, 7. Study methodology, 8. Number of participants, 9. Participants demographics, 10. Intervention protocol, 11. Primary outcome: mortality, 12. Secondary outcomes, see Table 2. The data was input into a table on Microsoft Excel (version 16.40).

### Risk of bias analysis

The risk of bias analysis of included studies was performed using the Cochrane risk-of-bias tool for randomised trials (RoB 2) [19]. This tool is structured into a fixed set of types of bias, each assessing different aspects of trial design, procedures and reporting. It is represented graphically with each study classified as low, unclear and high risk of bias, for each of the different types of biases. The risk of bias analysis was performed independently by reviewers. The modelling and graph was performed using Microsoft Excel (version 16.40).

### Data analysis

The primary and secondary outcomes were expressed as dichotomous and continuous outcomes; the dichotomous outcomes were reported as risk ratios (RR) with 95% confidence intervals (CI) following estimation using the Mantel–Haenszel method. Continuous variables were expressed as mean differences (MD) with 95% confidence intervals (CI) using the inverse variance method. Random effects modelling were applied for all primary and secondary outcomes, regardless of statistical heterogeneity (*I*^2^) value due to significant qualitative heterogeneity of the studies. All tests of significance were two tailed with *P* < 0.05 utilised to determine statistical significance. Meta-analysis was performed using Review Manager (RevMan) version 5.4.1 software, the Cochrane Collaboration 2020.

## Results

### Literature search

The systematic search strategy identified a total of 1497 studies; 576 duplicate records were subsequently removed. The remaining studies initially underwent title review, abstract review and full text review with 921, 256 and 45 reviewed respectively at each stage. Post full text review 19 studies were assessed for eligibility. After further exclusion of 5 studies, 14 remaining studies were determined to have satisfied the inclusion and exclusion criteria and were included in the systematic review and meta-analysis [12, 20–33]. The details of this are outlined in the PRISMA 2020 flowchart, see Table 4 [34].

**Table 4:**
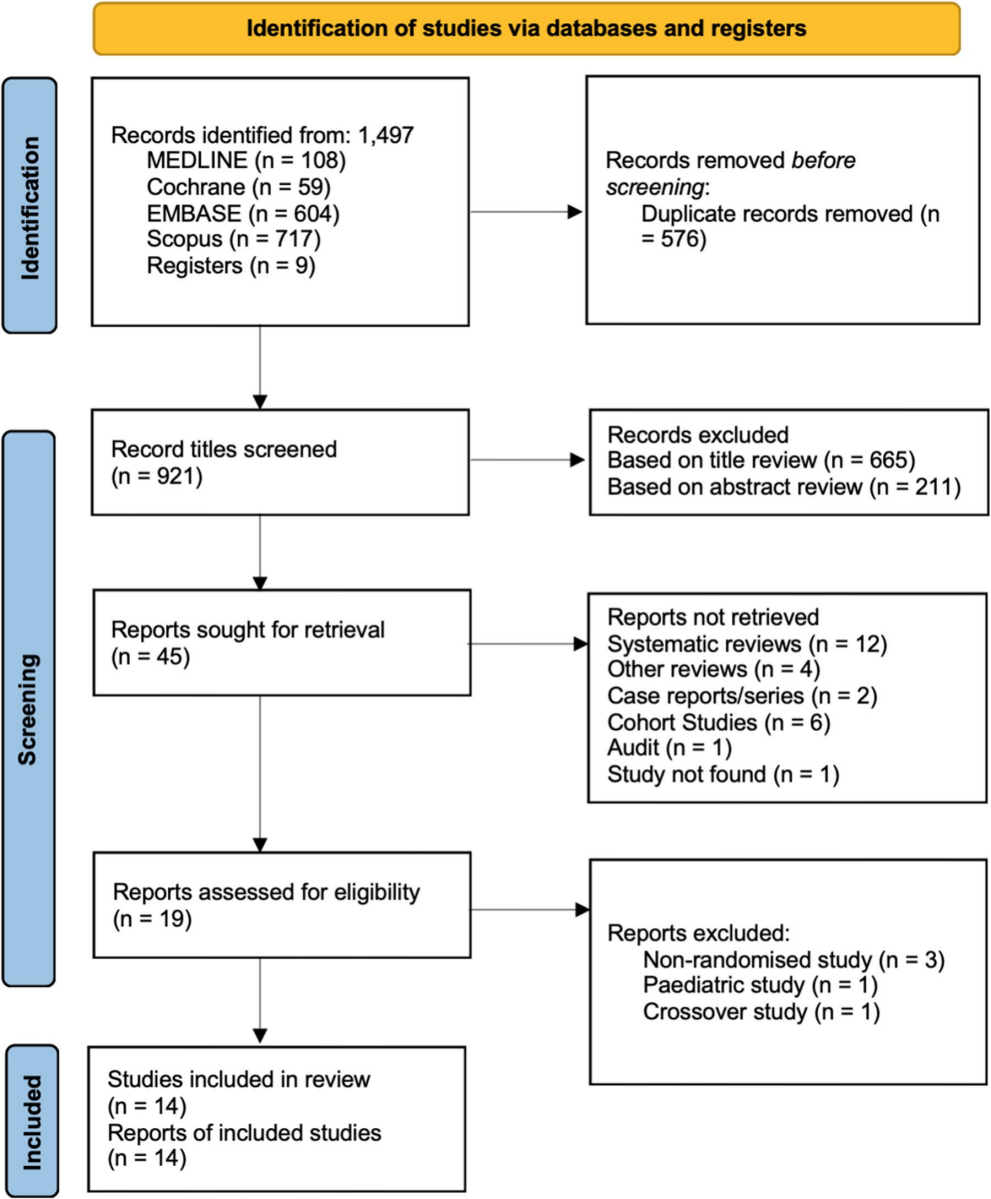
PRISMA 2020 flow diagram [34]

### Included study characteristics

Of the included 14 studies, there was marked geographical and chronological variation. They include 7 European, 4 Asian, 2 Australian and one large multinational trial (HALT-IT) with participants from 15 countries across the developed and developing world. The 14 studies included data from 14,338 patients, 34.93% were female (*n* = 5008), and 65.07% were male (*n* = 9330). The weighted mean age overall was 58.4 years with the mean of the TXA group being 58.34 years and the placebo group being 58.46 years. The year of publication of the trials spanned 48 years from Cormack et al. in 1973 to HALT-IT and Bashiri et al. in 2021 [12, 20–33]. While all 14 trials included placebo groups, there were significant variation in the intervention groups. TXA was administered through a variety of routes, at different doses and for different durations. See Appendix for details of individual included study characteristics. From the time of the first trial in 1973 to the last in 2021, there have been a number of notable changes to management of gastrointestinal bleeding, with the routine use of proton pump inhibitors (PPI) and the emergence of therapeutic endoscopic methods to control gastrointestinal bleeding [35]. For these reasons, it was decided that there was qualitatively significant heterogeneity between the trials and that random effect modelling should be used for meta-analysis calculation.

### Risk of bias assessment

The risk of bias for the included studies was evaluated using the Cochrane risk-of-bias tool for randomised trials (RoB 2) [19]. The evaluation of bias was independently carried out by two reviewers. The results of the evaluation are graphically represented in Table 5.

**Table 5:**
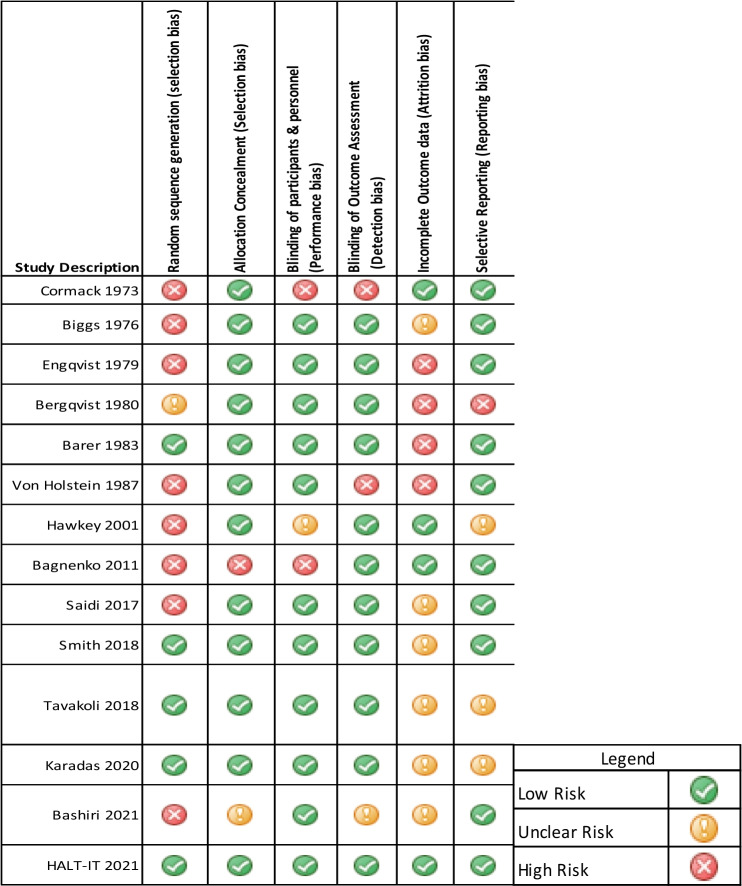
Risk of bias assessment table [19]

### Tabulated meta-analysis outcomes (Table 6)

**Table 6:** Tabulated meta-analysis outcomes

**Outcome**	**Studies**	**# of participants**	**Statistical method**	**Effect estimate**
**Mortality**	14	14,338	Risk ratio (M-H, random, 95% CI)	0.86 [0.74, 1.00]
**Rebleeding**	12	13,968	Risk ratio (M-H, random, 95% CI)	0.85 [0.71, 1.03]
**Need for intervention**	8	13,195	Risk ratio (M-H, random, 95% CI)	0.92 [0.76, 1.11]
**Therapeutic endoscopic intervention**	6	12,905	Risk ratio (M-H, random, 95% CI)	0.92 [0.70, 1.22]
**Surgical intervention**	12	13,944	Risk ratio (M-H, random, 95% CI)	0.75 [0.53, 1.07]
**Transfusion requirement**	10	13,343	Risk ratio (M-H, random, 95% CI)	1.01 [0.94, 10.7]
**Transfusion volume**	5	12,352	Mean difference (IV, random, 95%CI)	− 0.68 [1.51, 0.14]
**Length of stay**	4	12,595	Mean difference (IV, random, 95%CI)	0.03 [− 0.03, 0.08]
**ICU length of stay**	2	12,341	Mean difference (IV, random, 95%CI)	− 0.92 [− 2.26, 0.43]
**Venous thromboembolic events**	7	13,209	Risk ratio (M-H, random, 95% CI)	1.29 [0.53, 3.16]
**Arterial thromboembolic events**	5	12,777	Risk ratio (M-H, random, 95% CI)	0.94 [0.63, 1.40]

### Primary outcome: mortality

**Fig. 1:**
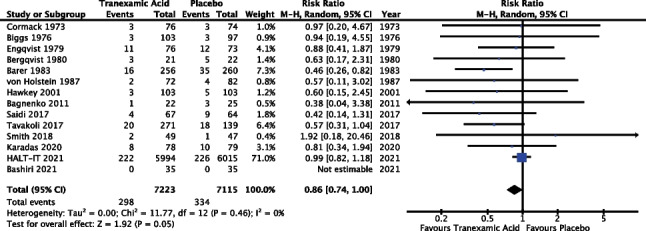
Meta-analysis forest plot assessing the effect of TXA versus placebo on mortality

All 14 trials reported outcomes for analysis in relation to mortality (100%). The meta-analysis included data from 14,338 patients. However, the results from Bashiri et al. (*n* = 70) were not estimable as there were no incidences of mortality in either the TXA or placebo groups [22]. Results of the meta-analysis (Fig. 1) revealed a risk ratio of 0.86 with 95% confidence interval ranged from 0.74 to 1.00 and *P* 0.05; as such the result did not reach statistical significance. The *I*^2^ value was 0%; however, random effects modelling was used as described above, see “Included study characteristics”.

### Secondary outcomes

#### Rebleeding rate

**Fig. 2:**
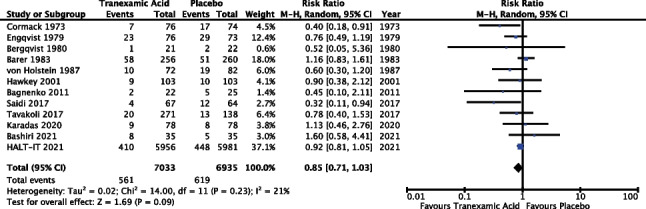
Meta-analysis forest plot assessing the effect of TXA versus placebo on rebleeding

Twelve of the 14 trials reported outcomes for analysis in relation to rebleeding rate (85.7%). The meta-analysis included data from 13,968 patients. Results of the meta-analysis (Fig. 2) revealed a risk of ratio of 0.85 with 95% confidence interval from 0.71 to 1.03 and *P* 0.09; as such the result did not reach statistical significance. The *I*^2^ value was 21%; however, random effects modelling was used as described above, see “Included study characteristics”.

#### Need for intervention

**Fig. 3:**
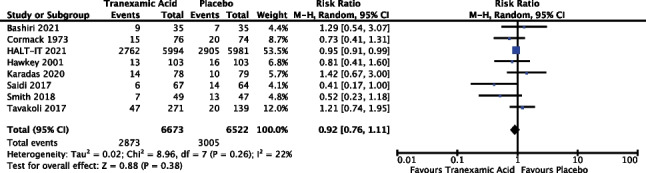
Meta-analysis forest plot assessing the effect of TXA versus placebo on the need for intervention

Eight of the 14 trials reported outcomes in relation to intervention (57.1%). Where specific intervention data was provided (therapeutic endoscopic intervention and surgical intervention), the data was analysed separately, see below meta-analysis for need for therapeutic endoscopic intervention and need for surgery. This meta-analysis includes data from 13,195 patients. Results of the meta-analysis (Fig. 3) reveal a risk ratio of 0.92 with a 95% confidence interval from 0.76 to 1.11 and *P* 0.38; as such the result did not reach statistical significance. The *I*^2^ value was 22%; however, random effects modelling was used as described above, see “Included study characteristics”.

#### Need for therapeutic endoscopic intervention

**Fig. 4:**
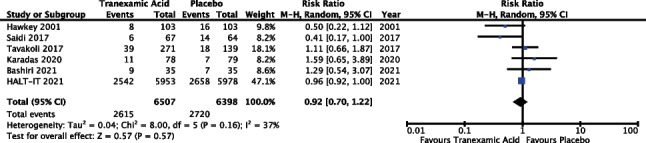
Meta-analysis forest plot assessing the effect of TXA versus placebo on the need for therapeutic endoscopic intervention

Six of the 14 trials reported outcomes in relation to therapeutic endoscopic intervention (42.9%). The meta-analysis included data from 12,905 patients. Results of the meta-analysis (Fig. 4) reveal a risk ratio of 0.92 with a 95% confidence interval from 0.7 to 1.22 and *P* 0.57; as such the result did not reach statistical significance. The *I*^2^ value was 37%; however, random effects modelling was used as described above, see “Included study characteristics”.

#### Need for surgical intervention

**Fig. 5:**
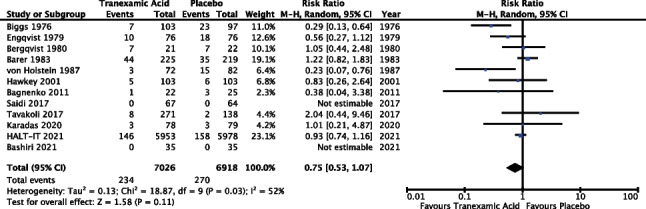
Meta-analysis forest plot assessing the effect of TXA versus placebo on the need for surgical intervention

Twelve of the 14 trials reported outcomes in relation to surgical intervention (85.7%). The meta-analysis included data from 13,944 patients. However, Saidi et al. (*n* = 131) and Bashiri et al. (*n* = 70) were not estimable as there were no incidences of surgical intervention in either the TXA or placebo groups in either trial [22, 36]. Results of the meta-analysis (Fig. 5) revealed a risk ratio of 0.75 with a 95% confidence interval from 0.53 to 1.07 and *P* 0.11; as such the result did not reach statistical significance. The *I*^2^ value was 52% and as such random effects modelling was used.

#### Transfusion requirement

**Fig. 6:**
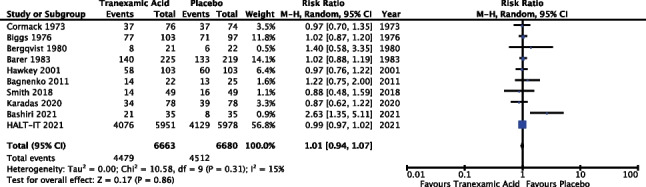
Meta-analysis forest plot assessing the effect of TXA versus placebo on transfusion requirement

Ten of the 14 trials reported outcomes in relation to transfusion requirement (71.4%). The meta-analysis included data from 13,343 patients. Results of the meta-analysis (Fig. 6) revealed a risk ratio of 1.01 with a 95% confidence interval from 0.94 to 1.07 and *P* 0.86; as such the result did not reach statistical significance. The *I*^2^ value was 15%; however, random effects modelling was used as described above, see “Included study characteristics”.

#### Transfusion volume

**Fig. 7:**
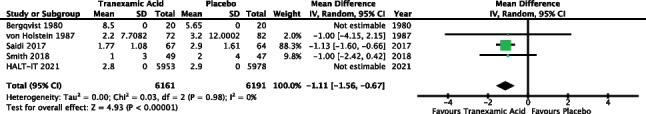
Meta-analysis forest plot assessing the effect of TXA versus placebo on transfusion volume

Five of the 14 trials reported outcomes in relation to mean transfusion volume (35.7%), expressed as units of packed red blood cells. The meta-analysis included data from 12,352 patients. However, data from Bergqvist et al. was not estimable as details of standard deviation or other variances metrics were not provided [20]. Results of the meta-analysis (Fig. 7) revealed a mean difference of − 0.68 with 95% confidence interval from − 1.51 to 0.14 and *P* 0.10; as such the result did not reach statistical significance. The *I*^2^ value was 84%; however, random effects modelling was used as described above, see “Included study characteristics”.

#### Overall length of stay

**Fig. 8:**
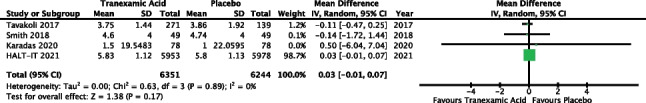
Meta-analysis forest plot assessing the effect of TXA versus placebo on overall length of stay

Four of the 14 trials reported outcomes in relation to mean length of stay as hospital inpatient (33.3%). The meta-analysis included data from 12,595 patients. Results of the meta-analysis (Fig. 8) reveal a mean difference of 0.03 with 95% confidence interval from − 0.03 to 0.08 and *P* 0.31; as such the result did not reach statistical significance. The *I*^2^ value was 0%; however, random effects modelling was used as described above, see “Included study characteristics”.

#### ICU length of stay

**Fig. 9:**
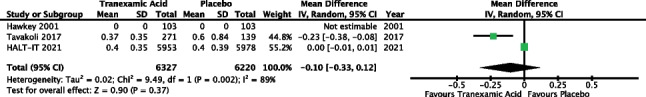
Meta-analysis forest plot assessing the effect of TXA versus placebo on ICU length of stay

Two of the 14 trials reported outcomes in relation to mean length of ICU stay (16.7%). Hawkey et al. reported proportion of patients requiring ICU stay but not the mean length and as such have been omitted from this meta-analysis [27]. Results of the meta-analysis (Fig. 9) reveal a mean difference of − 0.92 with 95% confidence interval from − 2.26 to 0.43 and *P* 0.18; as such the result did not reach statistical significance. The *I*^2^ value was 100%, and as such random effects modelling was used.

#### Venous thromboembolic events

**Fig. 10:**
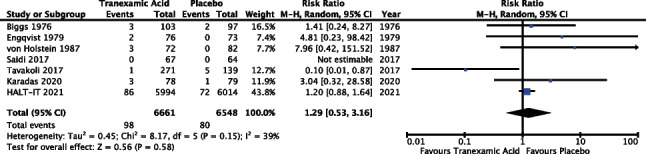
Meta-analysis forest plot assessing the effect of TXA versus placebo on venous thromboembolic events

Seven of the 14 trials reported outcomes in relation to venous thromboembolic events (50%), including deep venous thrombosis (DVT) and pulmonary embolism (PE). The meta-analysis included data from 13,209 patients; however, Saidi et al. (*n* = 131) were not estimable as there were no incidences of VTE in either TXA or placebo groups [36]. Results of the meta-analysis (Fig. 10) revealed a risk ratio of 1.29 with a 95% confidence interval from 0.53 to 3.16 and *P* 015; as such the result did not reach statistical significance. The *I*^2^ value was 39%; however, random effects modelling was used as described above, see “Included study characteristics”.

#### Arterial thromboembolic events

**Fig. 11:**
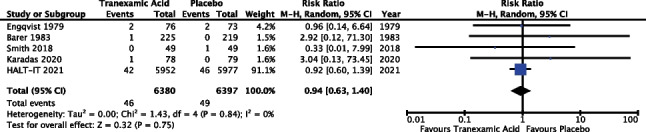
Meta-analysis forest plot assessing the effect of tranexamic acid versus placebo on arterial thromboembolic events

Five of the 14 trials reported outcomes in relation to arterial thromboembolic events (35.7%), including cerebrovascular accidents (CVA), transient ischaemic attacks (TIA) and myocardial infarctions (MI). The meta-analysis included data from 12,777 patients. Results of the meta-analysis (Fig. 11) revealed a risk ratio of 0.94 with a 95% confidence interval from 0.63 to 1.4 and *P* 0.75; as such the result did not reach statistical significance. The *I*^2^ value was 0%; however, random effects modelling was used as described above, see “Included study characteristics”.

#### Other adverse effects

There were a number of other adverse effects discussed in the 14 trials; however, there was insufficient data in order to perform a meta-analysis. The most commonly described adverse effect in the TXA group was GI upset. The other finding of note comes from the large HALT-IT trial which described a statistically significant increased risk of seizure in the TXA group when compared with the placebo group. HALT-IT detailed seizures in 38 patients (0.6%) in the TXA group versus 22 patients (0.4%) in the placebo group, with a risk ratio of 1.73 with 95% confidence interval of 1.03 to 2.93 [12]. It was not possible to perform a meta-analysis as the other trials had insufficient data on seizures reported.

#### Subgroup analysis

##### TXA on mortality in upper GI bleeding versus lower GI bleeding

**Fig. 12:**
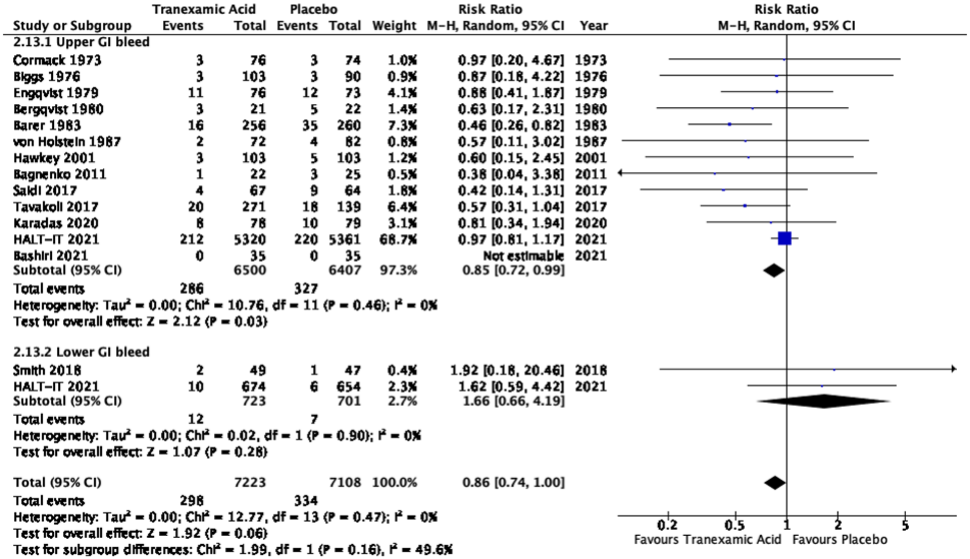
Subgroup meta-analysis of TXA versus placebo on mortality in upper and lower GI bleeding

The majority of trials exclusively studied the use of TXA in upper GI bleeding. HALT-IT and Smith et al. were the only 2 trials that included patients with lower GI bleeding. Subgroup analysis looking at mortality in patients with lower GI bleeding showed no statistically significant difference between TXA and placebo with a risk ratio of 1.66 with 95% confidence interval of 0.66 to 4.19 and 0.28. However, the subgroup meta-analysis of mortality in upper GI bleeding demonstrated a statistically significant difference between TXA and placebo with a risk ratio of 0.85 with 95% confidence interval of 0.72 to 0.99 and *P* 0.03 (Fig. 12).

##### TXA on mortality in variceal versus non-variceal bleeding

**Fig. 13:**
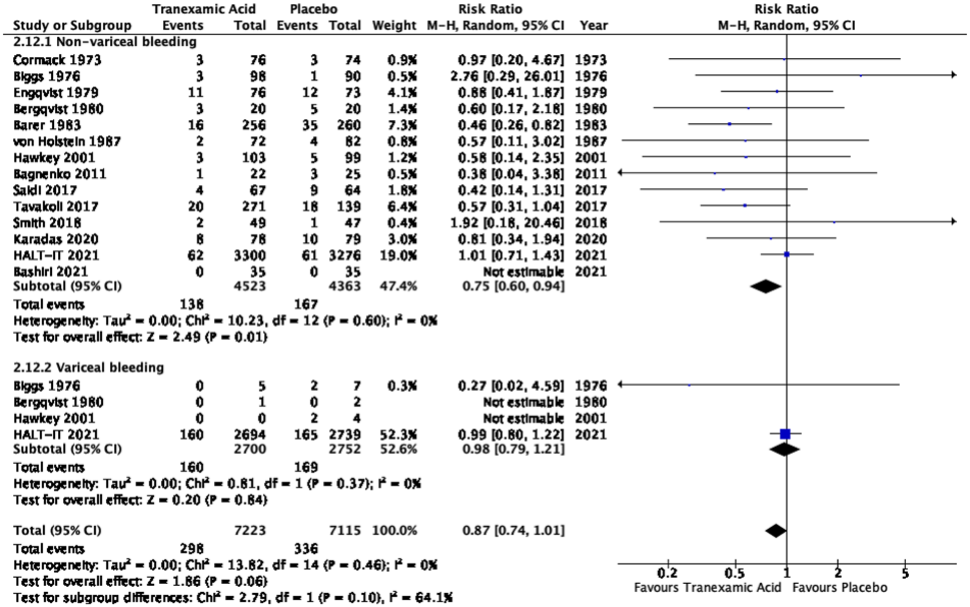
Subgroup meta-analysis of TXA versus placebo on mortality in non-variceal and variceal bleeding

Variceal bleeding data was not included in enough detail in many of the RCTs to facilitate adequate inclusion in the meta-analysis. Trials such as Bashiri et al., Karadas et al. and Saidi et al. excluded patients with variceal bleeding during initial patient selection. A number of studies (Bergqvist et al. and Hawkey et al.) included sufficient detail to be included but there were insufficient numbers in the variceal bleeding groups to be included in the meta-analysis. The subgroup meta-analysis of TXA versus placebo of non-variceal bleeding included data from 13 RCTs and it demonstrated a statistically significant result with a risk ratio of 0.75 with 95% confidence interval of 0.6 to 0.94 and *P* 0.01. Only 4 trials included sufficient data to be included in the subgroup meta-analysis of TXA versus placebo in variceal bleeding which did not show a statistically significant result; risk ratio 0.98 with 95% confidence interval of 0.79 to 1.21 and *P* 0.84 (Fig. 13).

## Discussion

The primary outcome of this systematic review and meta-analysis showed that tranexamic acid did not reduce mortality in patients with gastrointestinal bleeding when compared with placebo (RR: 0.86 95% CI (0.74–1.00), *P* = 0.05). This builds on the evidence detailed in previous meta-analyses by Burke et al. and Kamal et al. [13, 14]. Similar to these reviews, this meta-analysis was heavily influenced by the large HALT-IT trial (*n* = 12,009) which found no reduction in mortality with TXA when compared with placebo. Prior to HALT-IT, a Cochrane review by Bennett et al. in 2014 had concluded that TXA appears to have a beneficial effect on mortality but this was uncertain due to a high dropout rate in a number of the trials [37].

Rebleeding is important in the pathogenesis and natural history of gastrointestinal bleeding. It often marks the transition from a stable to an unstable patient and precedes terminal bleeding events resulting in mortality [38]. As such, a medication that could prevent rebleeding could be useful in reducing mortality, ICU admission and transfusion requirements. However, despite this theoretical support for TXA in preventing rebleeding, this meta-analysis found no statistically significant reduction in rebleeding rates when TXA was compared with placebo (RR: 0.83 95% CI (0.71–1.03), *P* = 0.09).

This meta-analysis also assessed the role of TXA in reducing the need for intervention in gastrointestinal bleeding. Where segregated data was available within the trials, we divided intervention into therapeutic endoscopic interventions and surgical intervention. Looking firstly at overall intervention, there was no statistically significant difference between TXA and placebo (RR: 0.92 95% CI (0.76–1.11), *P* = 0.038). Similarly there was no reduction in therapeutic endoscopic interventions (RR: 0.92 95% CI (0.7–1.22), *P* = 0.57) or surgical intervention (RR: 0.75 95% CI (0.53–1.07), *P* = 0.11) with TXA when compared with placebo. We also looked at interventional radiology (IR) guided embolisation but data on this was only reported in the HALT-IT trial. Similar to other interventions, there was no statistically significant risk reduction with TXA when compared with placebo (RR: 0.83 95% CI (0.61–1.13)).

From a theoretical perspective, TXA, as an antifibrinolytic agent, may prevent fibrinolysis and increase the risk of propagation and embolisation of clots and therefore increase the incidence of thromboembolic events (TE) [15]. In 2021, Taeuber et al. [39] performed a more extensive systematic review and meta-analysis which incorporated 216 trials and 125,550 patients. They found that thromboembolic events occurred in 2.1% (*n* = 1020) of the intravenous TXA group versus 2.0% (*n* = 900) in the control group. They found no association between intravenous TXA and thromboembolic events (risk difference: 0.001 95% CI (− 0.001–0.002), *P* = 0.49). Our meta-analysis looked at both arterial thromboembolic events (MI, CVA, TIA) and venous thromboembolic events (DVT, PE). Only 6 trials reported data on VTE, and 5 trials reported data in relation to arterial thromboembolic events. Despite the HALT-IT trial reporting increased risk of VTE [12], our meta-analysis found that there was no statistical significance between TXA with venous thromboembolic events (RR: 1.20 95% CI (0.90–1.60), *P* = 0.22) or arterial thromboembolic events (RR: 0.94 95% CI (0.63–1.40), *P* = 0.75) when compared with placebo. Our meta-analysis finding is consistent with the findings of Taeuber et al. in showing no association between TXA and thromboembolic events.

Of other adverse effects mentioned throughout the trials, nausea and vomiting was the most common. However, data in relation to this outcome was not provided in sufficient detail to facilitate a meta-analysis. Many trials included incidences of nausea and vomiting but either did not specify the breakdown of cases between TXA and placebo [20–23, 26, 27, 29, 31, 32, 36] groups or provided data on events in the TXA group with no comparative figures from the placebo group. The other adverse effect of note was seizures. While the majority of trials did not report incidence of seizures, HALT-IT reported a statistically significant increase risk of seizures in the TXA group when compared with placebo, with 38 events occurring in the TXA group compared with 22 in the placebo (0.6% vs 0.4%, RR 1.73 95% CI (1.03–2.93)) [12]. However, as highlighted by Murao et al. in their systematic review, the risk of seizures from TXA is dose dependent [40]. They noted that specifically doses of above 2 g of TXA per 24 h confer an increased risk of seizure. The TXA doses used for GI bleeding often exceeded this 2 g per 24 h, for example HALT-IT used 1 g stat and a further 3 g over 24 h in a continuous 125 mg/h infusion. The doses used in TXA for GI bleeding often exceeded the doses used in other large TXA trials, such as CRASH-2 (1 g stat and 1 g infused over 24 h) and WOMAN (1 g stat and further 1 g if recurrent bleeding). Therefore, the contraindication of TXA in patients with known seizure disorders may only be applicable when using higher doses of TXA, specifically when using fixed dose in patients suffering with renal impairment or liver disease.

The aim of this systematic review was to build on previous reviews performed by Burke et al. and Kamal et al. [13, 14] and to specifically include studies investigating lower gastrointestinal bleeding, similar to the HALT-IT trial [12]. However, this strategy only yielded 2 additional trials Smith et al. (*n* = 96) and Bashiri et al. (*n* = 70) [22, 31]. The meta-analysis outcomes of this review have not revealed any additional findings when compared with Kamal et al. [14].

Subgroup analysis was performed looking into whether TXA was beneficial in patients with upper GI bleeding, lower GI bleeding and whether the bleed aetiology was non-variceal or variceal bleeding. Subgroup analysis demonstrated that when upper GI bleed cases were isolated there was a statistically significant mortality risk reduction with TXA when compared with placebo, RR 0.85 95% CI (0.72–0.99). However, the weight of the HALT-IT RCT is diluted in this subgroup meta-analysis. HALT-IT is responsible for 82.7% of patients in the overall meta-analysis (10,681/12,914); however, its weighting in the meta-analysis is 68.7%.

It must also be noted that the trial HALT-IT is more rigorous in its methodology than the smaller, more biased trials. These two facts combined and the proximity of the upper margin of the confidence interval to 1 would lend doubt to the true significance of the subgroup meta-analysis findings. HALT-IT individually found no statistically significant difference in mortality in the upper GI bleeding subgroup. It is possible the segregation of upper and lower GI diluted the statistical power of HALT-IT allowing smaller, less methodologically rigorous trials to push the meta-analysis calculation towards statistical significance. It must also be noted that the well designed and conducted HALT-IT trial subgroup analysis clearly showed no statistical difference between the TXA and placebo groups both with a risk ratio close to 1 (0.97) and a confidence interval sitting equally either side of 1 from 0.81 to 1.17.

Subgroup analysis assessing the effect of TXA versus placebo on mortality in patients with non-variceal bleeding demonstrated a statistically significant risk reduction, RR 0.75 95% CI (0.60–0.94). Similar to the above criticism of the subgroup analysis for UGIB, the non-variceal bleeding subgroup meta-analysis dilutes the impact of the HALT-IT RCT. HALT-IT accounts for 74% (6576/8886) of patients but was only weighted at 19%. Also when looking at the subgroup analysis from the HALT-IT trial individually, it clearly shows no difference between the subgroups with risk reduction of 1.01, 95% CI (0.71–1.43).

Subgroup analysis looking into dosing or route of administration was not performed as part of this review. However, a systematic review and meta-analysis by Dionne et al. assessed the difference between high-dose and low-dose TXA for both upper and lower GI bleeding [41]. They found that high-dose IV TXA did not improve outcomes and conferred an increased risk of VTE and seizures. When looking at low-dose TXA, their meta-analysis showed no effect on mortality but did show a statistically significant risk reduction in rebleeding and need for surgery. Their findings contradict the expected dose–response relationship and, coupled with the somewhat arbitrary delineation of “low” versus “high” dose, it is uncertain whether there findings are of true significance.

Overall looking into the different subgroups, it is uncertain whether the statistically significant meta-analysis results hold up as truly significant on closer analysis. If anything can be taken from the subgroup analysis, it may be that future research or trials should focus more on the use of TXA in cases of non-variceal upper GI bleeding. This also has some pathophysiological support, through inhibition of gastric hyperfibrinolysis described by Cox et al. [7]. Future research should potentially focus on thromboelastography (TEG) to identify acute gastrointestinal bleeding patients with evidence of hyperfibrinolysis. TXA is likely to be more beneficial in this subset of patients.

This systematic review and meta-analysis has a number of limitations. These include the high risk of bias and high numbers of dropouts in a number of the older, smaller RCTs. There are also significant differences in the number of participants. The majority of the older, smaller trials are eclipsed in terms of participants by HALT-IT which accounts for 82.7% of the overall participants in the review. The chronology of the trials is also a limiting factor. The first trial included is Cormack et al. which was performed in 1973 with the latest trial, HALT-IT being performed in 2021. This duration, spanning 48 years, results in bias due to the significant changes in medical practice and the management of acute gastrointestinal bleeding over the time period.

There are a significant amount of non-randomised cohort studies, audits and case reports that looked at the use of TXA in gastrointestinal bleeding. Many of these, due to poor or biased study methodology or low participant numbers, do not provide robust outcomes that challenge the RCTs discussed in this systematic review. However, two studies merit mention.

The first is a large retrospective cohort study (*n* = 10,254) from Taiwan by Ting et al. published in 2022 [42]. The retrospective study used data from Taiwan’s National Health Insurance Research Database (NHIRD) to compare early administration of TXA versus late administration of TXA for gastrointestinal bleeding, with “early” being determined as within the ED and “late” being after being admitted from the ED. Multiple Cox regression analysis revealed significantly lower mortality in the early TXA versus the late TXA group (adjusted hazard ratio 0.64 95% CI (0.57–0.73)). It also reported no significant increase in thromboembolic events in early versus late TXA groups (adjusted hazard ratio 1.03 95% CI (0.94–1.12)). However, it must be noted that Ting et al. compared early versus late TXA administration instead of TXA versus control or TXA versus no TXA groups. Despite the number of participants in this cohort studies, due to the retrospective approach and cohort study methodology, it is more susceptible to biases than an RCT. Given the limitations and inherent biases of this type of study, it is uncertain that the results are sufficient to contradict the findings of the more methodologically rigorous randomised control trials, such as HALT-IT [12].

Another large Japanese retrospective observational study (*n* = 61,052) by Miyamoto et al. assessed the use of TXA for diverticular lower gastrointestinal bleeding and determined that there was no statistically significant difference in in-hospital mortality between the TXA and control group (OR: 1.07 95% CI (0.88–1.30)) [43]. They did find that the TXA group were less likely to develop severe bleeding (OR: 0.93 95% CI (0.89–0.99)) or require blood transfusions (OR: 0.88 95% CI (0.84–0.92)). The TXA group had shorter hospital stays − 0.23 days 95% CI (− 0.01–0.44) and cost of admission was also reduced (− 233 USD 95% CI (− 153 to − 314)). This study does not show a benefit in relation to mortality. When interpreting the secondary outcomes (decreases in severe bleeding and need for transfusion), again similar to Ting et al., it is important to note this trial’s limitations and biases when trying to compare with well conducted RCTs.

There are 6 ongoing trials assessing TXA in gastrointestinal bleeding with statuses of either unknown or recruiting on the EU Clinical Trials Registry and ClinicalTrials.gov registry, see Table 7. Two of these, EXARHOSE [44], a French study with an estimated enrolment of 500, and an Indian RCT with an enrolment target of 600, are specifically looking at the use of TXA in liver cirrhosis-related upper gastrointestinal bleeding. The total estimated number of patients to be enrolled in these upcoming trials is 2034. The status of some trials is unknown on the trial registers and therefore their outcomes may not be published. This includes HEXUGI, an Israeli trial commenced in 2014 (est. *n* = 300), and TAUGI, a South Korean trial commenced in 2012 (est. *n* = 414). It is difficult to ascertain the impact these ongoing trials may have in the debate on the use of TXA in gastrointestinal bleeding. It is unlikely that these trial results would greatly influence the field given the low enrolment numbers when compared with the already published trials, especially that of the large HALT-IT trial (*n* = 12,009).

**Table 7:** Recruiting or unknown status RCTs from clinical trial registers

**Title**	**Status (country and year)**	**Est. enrolment**	**Status**	**Reference**
The Use of Hexacapron in Upper Gastrointestinal Bleeding (HEXUGI)	Incomplete RCT (Israel 2014)	300	Unknown	NCT02071316
Precise Delivery of Tranexamic Acid to Enhance Endoscopic Hemostasis for Peptic Ulcer Bleeding	Ongoing unreported RCT (Taiwan 2022)	60	Recruiting	NCT05248321
Tranexamic Acid for Acute Upper Gastrointestinal Bleed in Cirrhosis	Ongoing unreported RCT (India 2020)	600	Recruiting	NCT04489108
Efficacity and Safety of Tranexamic Acid in Cirrhotic Patients Presenting With Acute Upper Gastrointestinal Bleeding (EXARHOSE)	Ongoing unreported RCT (France 2017)	500	Unknown	NCT03023189
Efficacy of Tranexamic Acid in Upper Gastrointestinal Bleeding	Ongoing unreported RCT (India 2021)	160	Recruiting	NCT04788121
Tranexamic Acid for Upper Gastrointestinal Bleeding (TAUGIB)	Ongoing unreported RCT (South Korea 2012)	414	Unknown	NCT01713101

## Conclusion

The primary outcome of this systematic review and meta-analysis showed that tranexamic acid did not reduce mortality in patients with upper or lower gastrointestinal bleeding when compared with placebo. Our study, the first to incorporate both upper and lower gastrointestinal bleeding together, also found no improvement in secondary outcomes (rebleeding, transfusion requirement, need for intervention or length of stay) when compared with placebo. We found no significant association between tranexamic acid and venous thromboembolic events. However, there appears to be an increased risk of seizures with tranexamic acid. Given the results of our meta-analysis, we would not recommend the routine use of tranexamic acid for patients presenting with acute gastrointestinal bleeding.

## Data Availability

The data that support the findings of this study are available from the corresponding author, [OO'D], upon reasonable request.

## Appendix

**Table:** **Table 8** Study characteristics table

**Author (year)**	**Country (centres)**	**Method**	**No**	**Intervention**	**Demographics**	**Female (%)**	**Mean age (years)**
**Cormack (1973)**	UK (single)	Unblinded randomised placebo-controlled trial	150	1.5 g TDS TCA (Cyklokapron) placebo tablets	Male 100 Female 50	33.3%	Not reported
**Biggs (1976)**	Australia (single)	Double blinded randomised placebo-controlled trial	200	Single dose 1 g IV followed by 1 g PO TDS for 5/7. Placebo	Intervention M 75 F 28 Control M 80 F 17	22.5%	Not reported
**Engqvist (1979)**	Sweden (single)	Double blinded randomised placebo-controlled trial	149	1 g IV 6 times daily × 3/7 then 1.5 g PO QDS × 4/7	Intervention M 55 F 21 Control M 61 F 12	22.1%	Intervention 59 Control 56
**Bergqvist (1980)**	Sweden (single)	Double blinded randomised placebo-controlled trial	43	2 g 6 times daily via NG × 2/7	Intervention M 14 F 7 Control M 20 F 2	20.9%	Intervention 61 Control 58
**Barer (1983)**	UK (two centres)	Double blinded randomised placebo-controlled trial	516	1 g IV QDS × 2/7 then 1 g PO QDS × 5/7	Intervention M 177 F 79 Control M 155/105	35.7%	Intervention 60 Control 63
**Von Holstein (1987)**	Sweden (single)	Double blinded randomised placebo-controlled trial	154	1 g IV 6 times daily × 3/7 then 1.5 g PO QDS × 3/7	Intervention M 50 F 22 Control M 58 F 24	29.9%	Intervention 62 Control 65
**Hawkey (2001)**	UK (4 centres)	Double blinded randomised placebo-controlled trial	414	2 g loading dose then 1 g QDS ± lansoprazole versus placebo	TXA M 69 F 34 Control M 67 F 36	33.8%	Intervention 58 Control 58
**Bagnenko (2011)**	Russia (single)	Open randomised trial (TXA vs no intervention)	47	10 mg/kg IV/PO TDS × 3/7	Male 29 Female 18	38.3%	Intervention 62 Control 64
**Saidi (2017)**	Iran (single)	Double blinded randomised placebo-controlled trial	131	1 g single dose via NG	Intervention M 41 F 26 Control M 41 F23	37.4%	Intervention 64 Control 65
**Smith (2018)**	Australia (single)	Double blinded randomised placebo-controlled trial	96	1 g QDS PO × 4/7 (renal dosing PRN)	Intervention M 32 F 17 Control M 31 F 16	34.4%	Intervention 68 Control 68
**Tavakoli (2018)**	Iran (single)	Double blinded randomised placebo-controlled trial	410	Regimen A: 1 g IV QDS Regimen B: 1 g IV QDS + 1 g single dose via NG	Regimen A: M 85 F 43 Regimen B: M 97 F 36 Control: M 92 F 47	33.2%	Regimen A: 61 Regimen B: 57 Control: 59
**Karadas (2020)**	Turkey (single)	Double blinded randomised placebo-controlled trial	157	Single dose 2 g 5% TXA in 100 ml NaCl via NG vs placebo 100 ml NaCl	Male 106 Female 51	32.5%	Intervention 63 Control 63
**Bashiri (2021)**	Iran (single)	Double blinded randomised placebo-controlled trial	70	1 g IV TDS	Intervention M 24 F 11 Control M 25 F 10	30.0%	Intervention 54 Control 55
**HALT-IT (2021)**	International (multi)	Double blinded randomised placebo-controlled trial	12,009	1 g TXA in 100 ml NaCl IV loading and 125 mg/h 24 h IV infusion	Intervention M 3852 F 2142 Control M 3891 F 2124	35.5%	Intervention 58 Control 58

## Abbreviations

CI: Confidence intervals
CVA: Cerebrovascular accident
DVT: Deep vein thrombosis
ED: Emergency Department
EACA: Epsilon-aminocaproic acid
GI: Gastrointestinal
GDPR: General Data Protection Regulation
LOS: Length of stay
LGIB: Lower GI bleeding
MD: Mean differences
MeSH: Medical Subject Headings
MH: Mantel-Haenszel
MI: Myocardial infarction
PE: Pulmonary embolism
PICO: Population, Intervention, Comparison and Outcome
PRISMA: Preferred Reporting Items for Systematic Reviews and Meta-Analyses
RCT: Randomised control trials
RR: Risk ratio
TIA: Transient ischemic attack
TXA: Tranexamic acid
UGIB: Upper gastrointestinal bleeding
VTE: Venous thromboembolism

## References

[1] El-Tawil AM (2012). Trends on gastrointestinal bleeding and mortality: where are we standing?. World J Gastroenterol.

[2] van Leerdam ME (2008). Epidemiology of acute upper gastrointestinal bleeding. Best Pract Res Clin Gastroenterol.

[3] Javaid T, Siddiqui N, Hasan S, Khan Z, Tabassum S, Khuder S et al (2016) The in-hospital mortality rate in gastrointestinal hemorrhage with shock: a nationwide analysis: 966. Am J Gastroenterol 111

[4] Dworzynski K, Pollit V, Kelsey A (2012). Management of acute upper gastrointestinal bleeding: summary of NICE guidance. BMJ.

[5] Watts G (2016). Utako Okamoto. Lancet.

[6] Roberts I, Shakur H, Coats T (2013). The CRASH-2 trial: a randomised controlled trial and economic evaluation of the effects of tranexamic acid on death, vascular occlusive events and transfusion requirement in bleeding trauma patients. Health Technol Assess.

[7] Cox HT, Poller L, Thomson JM (1967). Gastric fibrinolysis. A possible aetiological link with peptic ulcer. Lancet.

[8] Nilsson IM, Bergentz SE, Hedner U, Kullenberg K (1975). Gastric fibrinolysis. Thromb Diath Haemorrh.

[9] Shakur H, Elbourne D, Gülmezoglu M (2010). The WOMAN Trial (World Maternal Antifibrinolytic Trial): tranexamic acid for the treatment of postpartum haemorrhage: an international randomised, double blind placebo controlled trial. Trials.

[10] CRASH-3 trial collaborators (2019) Effects of tranexamic acid on death, disability, vascular occlusive events and other morbidities in patients with acute traumatic brain injury (CRASH-3): a randomised, placebo-controlled trial. Lancet 394(10210):1713-1723. 10.1016/S0140-6736(19)32233-0. Epub 2019 Oct 14. Erratum in: Lancet. 2019 Nov 9;394(10210):1712. PMID: 31623894; PMCID: PMC6853170 10.1016/S0140-6736(19)32233-0 PMC6853170 31623894

[11] Lin ZX, Woolf SK (2016). Safety, efficacy, and cost-effectiveness of tranexamic acid in orthopedic surgery. Orthopedics.

[12] Roberts I, Shakur-Still H, Afolabi A (2021). A high-dose 24-hour tranexamic acid infusion for the treatment of significant gastrointestinal bleeding: HALT-IT RCT. Health Technol Assess.

[13] Burke E, Harkins P, Ahmed I (2021). Is there a role for tranexamic acid in upper GI bleeding? A systematic review and meta-analysis. Surg Res Pract.

[14] Kamal F, Khan MA, Lee-Smith W (2020). Efficacy and safety of tranexamic acid in acute upper gastrointestinal bleeding: meta-analysis of randomised controlled trials. Scand J Gastroenterol.

[15] Ng W, Jerath A, Wąsowicz M (2015). Tranexamic acid: a clinical review. Anaesthesiol Intensive Ther.

[16] Moher D, Liberati A, Tetzlaff J, Altman DG (2009). Preferred reporting items for systematic reviews and meta-analyses: the PRISMA statement. BMJ.

[17] O’Donnell O, Regan M (2022) Systematic review of the use of tranexamic acid (TXA) in acute gastrointestinal bleeding. PROSPERO 2022. PROSPERO International prospective register of systematic reviews. Reference CRD42022308878

[18] Richardson WS, Wilson MC, Nishikawa J, Hayward RS (1995). The well-built clinical question: a key to evidence-based decisions. ACP J Club.

[19] Sterne JAC, Savović J, Page MJ (2019). RoB 2: a revised tool for assessing risk of bias in randomised trials. BMJ.

[20] Bagnenko SF, Verbitskiĭ VG (2011). Antifibrinolitic therapy for the treatment of massive ulcerative gastro-intestinal bleedings. Khirurgiia (Mosk).

[21] Barer D, Ogilvie A, Henry D (1983). Cimetidine and tranexamic acid in the treatment of acute upper-gastrointestinal-tract bleeding. N Engl J Med.

[22] Bashiri H, Hamzeii M, Bozorgomid A (2021). Effect of tranexamic acid on the treatment of patients with upper gastrointestinal bleeding: a double-blinded randomized controlled clinical trial. J Acute Dis.

[23] Bergqvist D, Dahlgren S, Hessman Y (1980). Local inhibition of the fibrinolytic system in patients with massive upper gastrointestinal hemorrhage. Upsala J Med Sci.

[24] Biggs JC, Hugh TB, Dodds AJ (1976). Tranexamic acid and upper gastrointestinal haemorrhage—a double-blind trial. Gut.

[25] Cormack F, Jouhar AJ, Chakrabarti RR, Fearnley GR (1973). Tranexamic acid in upper gastrointestinal haemorrhage. Lancet.

[26] Engqvist A, Broström O, Feilitzen FV (1979). Tranexamic acid in massive haemorrhage from the upper gastrointestinal tract: a double-blind study. Scand J Gastroenterol.

[27] Hawkey GM, Cole AT, McIntyre AS (2001). Drug treatments in upper gastrointestinal bleeding: value of endoscopic findings as surrogate end points. Gut.

[28] Hollanders D, Thomson JM, Schofield PF (1982). Tranexamic acid therapy in ulcerative colitis. Postgrad Med J.

[29] Karadas A, Dogan NÖ, Pinar SG et al (2020) A randomized controlled trial of the effects of local tranexamic acid on mortality, rebleeding, and recurrent endoscopy need in patients with upper gastrointestinal hemorrhage. Eur J Gastroenterol Hepatol 32(1) 10.1097/MEG.0000000000001555 31567714

[30] Rafeey M, Shoaran M, Ghergherechi R (2016). Topical tranexamic acid as a novel treatment for bleeding peptic ulcer: a randomised controlled trial. Afr J Paediatr Surg.

[31] Smith SR, Murray D, Pockney PG et al (2018) Tranexamic acid for lower GI hemorrhage: a randomized placebo-controlled clinical trial. Dis Colon Rectum 61(1) 10.1097/DCR.0000000000000943 29215478

[32] Tavakoli N, Mokhtare M, Agah S (2017). Comparison of the efficacy of intravenous tranexamic acid with and without topical administration versus placebo in urgent endoscopy rate for acute gastrointestinal bleeding: a double-blind randomized controlled trial. United European Gastroenterol J.

[33] von Holstein CC, Eriksson SB, Källén R (1987). Tranexamic acid as an aid to reducing blood transfusion requirements in gastric and duodenal bleeding. Br Med J (Clin Res Ed).

[34] Page MJ, McKenzie JE, Bossuyt PM (2021). The PRISMA 2020 statement: an updated guideline for reporting systematic reviews. BMJ.

[35] Laine L, McQuaid KR (2009). Endoscopic therapy for bleeding ulcers: an evidence-based approach based on meta-analyses of randomized controlled trials. Clin Gastroenterol Hepatol.

[36] Saidi H, Shojaie S, Ghavami Y (2017). Role of intra-gastric tranexamic acid in management of acute upper gastrointestinal bleeding. IIOAB J.

[37] Bennett C, Klingenberg SL, Langholz E, Gluud LL (2014). Tranexamic acid for upper gastrointestinal bleeding. Cochrane Database Syst Rev.

[38] García-Iglesias P, Villoria A, Suarez D (2011). Meta-analysis: predictors of rebleeding after endoscopic treatment for bleeding peptic ulcer. Aliment Pharmacol Ther.

[39] Taeuber I, Weibel S, Herrmann E (2021). Association of intravenous tranexamic acid with thromboembolic events and mortality: a systematic review, meta-analysis, and meta-regression. JAMA Surg.

[40] Murao S, Nakata H, Roberts I, Yamakawa K (2021). Effect of tranexamic acid on thrombotic events and seizures in bleeding patients: a systematic review and meta-analysis. Crit Care.

[41] Dionne JC, Oczkowski SJW, Hunt BJ (2022). Tranexamic acid in gastrointestinal bleeding: a systematic review and meta-analysis. Crit Care Med.

[42] Ting KH, Shiu BH, Yang SF et al (2022) Risk of mortality among patients with gastrointestinal bleeding with early and late treatment with tranexamic acid: a population-based cohort study. J Clin Med 11(6) 10.3390/jcm11061741 PMC8951209 35330064

[43] Miyamoto Y, Ohbe H, Ishimaru M (2021). Effect of tranexamic acid in patients with colonic diverticular bleeding: a nationwide inpatient database study. J Gastroenterol Hepatol.

[44] Heidet M, Amathieu R, Audureau E (2018). Efficacy and tolerance of early administration of tranexamic acid in patients with cirrhosis presenting with acute upper gastrointestinal bleeding: a study protocol for a multicentre, randomised, double-blind, placebo-controlled trial (the EXARHOSE study). BMJ Open.

